# The Home Learning Environment as a Mediator of the Impact of Parental Psychological Distress on Child Development

**DOI:** 10.3390/children13050582

**Published:** 2026-04-22

**Authors:** Marie-Louise (Jessica) A. J. van de Grint-Stoop, Laurel A. Fish, Chloe Austerberry, Marialivia Bernardi, R. M. Pasco Fearon

**Affiliations:** 1 Centre for Child, Adolescent and Family Research, Department of Psychology, University of Cambridge, Rayleigh Wing, Free School Lane, Cambridge CB2 3RF, UK; cmda2@cam.ac.uk; 2 Department of Clinical, Educational and Health Psychology, University College London, Gower Street, London WC1E 6BT, UK; l.fish@ucl.ac.uk (L.A.F.); m.bernardi@ucl.ac.uk (M.B.)

**Keywords:** psychosocial stimulation, millennium cohort study, parent mental health

## Abstract

**Highlights:**

**What are the main findings?**

- The home learning environment at 3 years significantly mediates associations between parental depressive symptoms at 9 months and children’s cognitive, language, and socio-emotional outcomes at age 5.
- Postnatal mental health in both mothers and fathers has significant indirect effects on child developmental outcomes through the home learning environment, but these indirect pathways are stronger for mothers.

**What are the implications of the main findings?**

- The impact of the positive parenting contributions, including the home learning environment, should be further investigated in the context of parental depression using quality-informing measurement instruments.
- Research and interventions on parental mental health should widen their focus beyond negative parenting behaviours to include positive, learning-oriented interactions.

**Abstract:**

**Background**: Research on the well-established association between maternal mental health problems and poorer child outcomes has focused on negative parenting behaviour and overlooked psychosocial stimulation as a potential mediating mechanism. Additionally, whether the same association exists for fathers has been understudied. **Methods**: We addressed these gaps using data from the nationally representative UK-based Millennium Cohort Study, including *n* = 15,623 children and their mothers (*n* = 14,922) and fathers (*n* = 12,408). Parental mental health and the home learning environment (HLE) were measured using the parent-reported Rutter Malaise Inventory at 9 months of age and the HLE Index at age 3, respectively. At 5 years of age, socio-emotional functioning was measured using the parent-reported Strengths and Difficulties Questionnaire, and cognitive and language abilities were assessed directly using British Ability Scales subtests. **Results**: Structural equation modelling indicated that the HLE significantly mediated the negative associations between PMH and children’s cognitive abilities (mother: β = −0.01, 95% CI [−0.01, −0.01], *p* < 0.001; father: β = −0.004, 95% CI [−0.008, −0.001], *p* = 0.025), socio-emotional functioning (mother: β = −0.01, 95% CI [−0.01, −0.01], *p* < 0.001; father, β = −0.004, 95% CI [−0.007, −0.001], *p* = 0.022), and language skills (mother: β = −0.01, 95% CI [−0.01, −0.01], *p* < 0.001; father: β = −0.005, 95% CI [−0.010, −0.001], *p* = 0.020). **Conclusions**: These findings support our hypotheses, with stronger associations identified for mothers than for fathers. The findings suggest that further research is needed on the impact of positive parenting, including the home learning environment, in the context of parental depression, using measurement instruments that provide insight in the quality of positive parenting.

## 1. Introduction

Extensive evidence shows that maternal mental health symptoms, and symptoms of depression in particular, are associated with poorer childhood development outcomes across the domains of cognitive, language, and motor skills, as well as adaptive behaviour [1,2]. However, our understanding of the mechanisms by which these developmental impacts arise remains limited [3,4]. Understanding mechanisms is particularly important in light of growing evidence that interventions designed to support child development in the context of postnatal depression have had limited success in improving child development outcomes [5].

Most accounts of the role of parenting in the negative effects of parental mental health problems on child development focus on parenting styles, such as ineffective or harsh discipline practices or qualitative features of parent–child interactions, such as parental insensitivity, unresponsiveness or poor parent–child relationship quality [6]. However, less attention has been paid to the potential importance of positive parental learning-oriented practices, such as stimulation and cognitively enriching parenting behaviours, captured in the concept of the home learning environment (HLE). Bradley [7] argues that although parents and their parenting behaviours are partly shaped by external factors such as socio-economic family characteristics [8], parents also actively try to shape the child’s environment to provide the child with stimulation, both physically, for example by providing toys and books, and through their actions and behaviours, such as engaging in positive learning-focused interactions such as playing with toys, reading stories or singing songs or rhymes [9]. A large number of studies has shown that these active learning, provisioning and stimulation behaviours are robustly associated with children’s language and cognitive development [10,11,12]. However, much less research has investigated whether they are negatively impacted by parental depression, and, critically, whether they mediate the association between parental depression and children’s developmental outcomes. In the current paper, we leverage large-scale nationally representative longitudinal data and well-validated direct assessments of child development to address both of these important and previously neglected questions in developmental research.

### 1.1. Cognitive Stimulation and the Home Learning Environment

The existing literature uses a variety of different terms to refer to parenting behaviours related to the provision of enriching, learning-, and development-oriented interactions and experiences [13,14]. These parental behaviours and practices are quite distinct from traditional social-learning or attachment-influenced parenting frameworks and constructs, such as authoritative/authoritarian parenting, inconsistent or harsh disciplinary practices or caregiver sensitive responsiveness [15]. Commonly used terms include cognitive stimulation, psychosocial stimulation, parental stimulation, or the home learning environment. These various terms all refer to broadly overlapping behaviours, typically including activities such as reading to the child, singing, telling stories, pretend play, or learning about shapes, letters, and number. Research on the role of these parental practices has its origin in the work of Caldwell and Bradley and in the observational measure, the Home Observation Measurement of the Environment (HOME) scale [16,17]. Less labour-intensive alternatives followed, building on the HOME, such as the interviewer-reported StimQ2 [18,19]. The HOME and its scales that specifically relate to the provision of learning opportunities have been used extensively in research on the effects of poverty on child development in many Western high-income countries [20,21]. Furthermore, stimulating parenting practices have also been assessed extensively in studies of parenting and child development in low- and middle-income countries in the global south [22], often using the family care indicators (FCIs), an instrument developed by UNICEF [23]. Building on this large body of observational research, a range of interventions have been developed that focus on supporting parents to increase children’s opportunities for early learning through the provision of toys and books, shared reading, interactive games and songs, and guidance for parents to create a language-rich environment for the child [24,25]. There is considerable evidence that these interventions are effective in increasing these parental behaviours and improving early cognitive and language development in both high and low-income countries [13]. In this paper we use the term ‘home learning environment’ or ‘HLE’ [26] to refer to these learning-oriented positive parenting practices.

### 1.2. The Home Learning Environment and Child Development

Previous studies have identified both short- and long-term effects of the early HLE. For example, significant short-term associations have been found between the HLE at age three and children’s language, cognition, and socio-emotional development at age 5, even when controlling for family background characteristics such as mother’s educational level and SES [26,27,28]. Hoyne and Egan [29] found that higher HLE scores (on a scale including items such as book reading, drawing, and painting) at age 3 were positively associated with higher vocabulary and non-verbal reasoning scores. Parents’ involvement in home learning activities at age 3 was also a significant contributor to differences in social development at the start of primary school [26]. Moreover, the size of the association between the HLE Index and child outcomes was larger than that of other family characteristics, such as mothers’ educational level [26]. Sylva and colleagues [26] also identified sex differences in the frequency of HLE activities. Parents of girls were significantly more likely than parents of boys to report that their children engage in activities such as reading, drawing and teaching nursery rhymes and songs. However, these sex differences did not affect the predictive relationship between the HLE and outcomes [26].

Beyond its short-term associations, the HLE Index has also been found to be a strong predictor of long-term educational attainment. Data from the nationally representative Millennium Cohort Study indicate that the HLE Index at age three predicts GCSE scores at age 16, after controlling for background characteristics such as SES [30]. Similarly, findings from the EPPSE study showed that children with higher age three HLE scores were more than three times as likely to take A-levels—63.5% of those with a score above 33/49, compared to just 18% of those with scores below 14/49 [31,32]. Overall, the longitudinal associations between the early HLE and cognitive, social and language development are robust and persist over time, even after the effects of other early predictors, such as early education, are controlled for.

### 1.3. Parental Mental Health, the Home Learning Environment and Early Childhood Development

There is extensive evidence of links between parental mental health problems and early childhood development across the domains of cognitive and language development and children’s emotional and behavioural difficulties [33,34,35,36]. A meta-analysis including over 190 studies found that mothers antenatal and postnatal mental health was associated to a broad range of child development domains, with the strongest associations for the domains specified above [1]. Effects of paternal postnatal depression points in the same direction as for mothers showing associations with later emotional and behavioural problems [37,38] and cognitive and language development [35].

There is some limited data suggesting the home learning environment as a potential mechanism explaining the association between parental depression and child development outcomes. For example, using data from the Millennium Cohort Study, Kiernan and Huerta [39] found that mothers who experienced depression symptoms when their baby was 9 months old engaged less often in reading activities when the child was aged 3 years, which was also associated cross-sectionally with the child’s cognitive development and emotional wellbeing. This provides some indication that mothers’ stimulating parenting behaviours might be affected over time by their mental state, but this study’s narrow focus on reading likely underestimates the impact. Furthermore, the cross-sectional data on reading and outcomes at age 3 does not allow a robust test of mediation.

While the primary focus of this paper is on the association between mothers’ mental health and early childhood development, we also explore the role of fathers’ mental health. Recent research has highlighted the importance and relative neglect of paternal postnatal depression [40]. Although more sparse, there is accumulating evidence that paternal postnatal depression also impacts children’s development. For example, data from the Avon Longitudinal Study of Parents and Children (ALSPAC) indicates reliable associations between fathers’ postnatal depression and psychiatric disorders in children 7 years later (independently of maternal postnatal depression; [38]). A recent systematic review on paternal mental distress including 48 cohorts indicated associations with general, cognitive, socio-emotional and language development [35]. Less is known about the role of fathers’ parenting in mediating the effects of paternal depression on child development. There are some indications that the associations between paternal depression and child development is less robust than maternal depression. For example, in one study poorer mental health in both mothers and fathers was associated with lower attainment in literacy, numeracy, language and emotional development at age 5 years, but only the effects of mothers’ mental health persisted when education and SES were controlled for [3].

### 1.4. The Current Study

In light of the above, this study investigated the following hypotheses:

(1) Higher parent psychological distress in the infant’s first year of life (age 9 months) predicts a lower home learning environment at age 3 years?
(2) Higher parent psychological distress in the infant’s first year of life (age 9 months) predicts worse developmental outcomes at age 5 years?
(3) The home learning environment at age 3 years mediates the association between parental psychological distress in infancy and developmental outcomes at age 5 years.

The present study focuses on age 5 outcomes, as this is a key stage coinciding with the start of formal schooling in the UK, when developmental outcomes are thought to be strongly shaped by the home environment [41]. We explore outcomes in the primary domains of child development—language, cognitive and socio-emotional development [42,43] in light of evidence connecting each of these individually to parental depression [33,34]. Although some studies combine cognitive and language development into a single construct, we treated them as distinct in order to explore whether each is uniquely impacted by aspects of the HLE, in particular because many of the HLE activities, such as shared reading, are inherently language-based. Our primary hypotheses and analyses focused on mothers’ depression symptoms, but we followed up these analyses by investigating whether the same pathways replicated in relation to fathers’ mental health. Given the more limited literature on paternal mental health, we expected similar negative associations, but treated these as exploratory.

## 2. Methods

### 2.1. Sample

We used longitudinal data from the Millennium Cohort study, a nationally representative UK birth cohort of over 19,000 children starting in the early 2000s. The data used for the current analysis were collected in 2001, 2004 and 2006, when the cohort children were 9 months, 3 and 5 years old. Families were included if there were data from at least one respondent (either the main respondent or partner respondent) when their child was 9 months, 3 years old or 5 years old, resulting in 15,623 (unweighted) included children. The sample was drawn from eligible families for Child benefits from the HMRC (His Majesty Revenue and Customs), for children born between 1 September 2000 and 31 August 2001 (England and Wales) and 24 November 2000 and 11 January 2002 (Scotland and Northern Ireland), alive and living in the UK at age 9 months (for more details on sampling see the technical report; [44]). The majority (*n* = 14,931) were recruited in the first sweep of data collection when the children were 9 months old and a small number of families (*n* = 692) entered this study at the second sweep of data collection, when the children were 3 years old because their addresses were unknown at the time, but they were eligible at sweep 1 [44]. Additional information on the sampling design is available in Supplementary Materials S1 [45,46]. From the selected sample, 50.75% were boys. There were 14,939 main respondents (see Footnote 1; 99.8% mothers), and 12,420 partners (see Footnote 1; 99.90% fathers). In total 14,932 mothers and 12,425 fathers were included in the analyses. Most cohort children (86.55%) and parents (mothers: 88.87%; fathers: 90.66%) were White. See Table 1 for a full breakdown of the cohort members’, mothers’ and fathers’ ethnicities, including sampling and non-response weights.

### 2.2. Measurements

#### 2.2.1. Parental Psychological Distress

The short form Rutter Malaise inventory was used to measure parental psychological distress, when the cohort child was nine months old [47], which included 9 out of the original 24 questions [48,49]. The inventory includes items on the two main components of psychological distress; depression and anxiety. Each item is score 0 (absent) or 1 (present) and a total sum score was computed. A score of 4 points or higher indicates the individual is experiencing symptoms associated with depression [48]. It is important to note that the Rutter Malaise Inventory is a self-report screening tool to measure psychological distress, but not a diagnostical tool and scores therefore do not reflect clinical depression. Overall, 13% of mothers scored 4 or higher, and 30% had a score of zero. For fathers 9% scored 4 or higher and 36% scored zero There was a relatively small correlation between maternal and paternal mental health *r* = 0.25, but significant, *p* < 0.001. Supplement S2 [50,51] contains details of confirmatory factor analysis (CFA) and exploratory factor analysis of the Rutter Malaise Inventory, confirming their unifactorial structure.

To address the extent to which any observed effects of depression during infancy were driven by continuity in depression scores over time, we also included that parent’s later mental health scores at three years of age using the Kessler 6 scale [52,53]. The Kessler 6 scale assesses the respondent’s psychological distress over the last 30 days, asking them how often they had felt depressed, hopeless, restless, or fidgety, whether they felt that everything they did required effort, and how often they felt worthless and nervous. Items were scored on a 5-point Likert from *All of the time* (4) to *None of the time* (0).

#### 2.2.2. Home Learning Environment

The home learning environment was measured using the HLE Index [54]. Supplement S3 [27,55,56] contains more details on the development of the scale and its use in MCS, and the same procedures were followed to create a total HLE Index score per cohort child. When the cohort children were 3 years old, six of the seven activities of the HLE Index were included in the parent questionnaire [54]. The main parent (98.5% mothers) in this study was asked to complete the HLE Index. All questions asked about the frequency with which *anyone* in the household did the following activities with the child: reading to their child, taking the child to the library, paining or drawing with the child, playing with numbers with the child, teaching the child letters, counting, and singing songs, poems and rhymes. Answers were scored on a 8-point Likert scale from *Never* (0) to *Multiple times a day* (7), except for the item on taking the child to the library, which was scored on a 5-point scale but recoded to match the scaling of the other items (see Supplement S3 [27,55,56] for details). Responses were summed to create a total HLE Index score ranging from 0 to 49. The internal consistency of the 7 items in our sample was fairly low (α = 0.59), likely due, in part, to the small number of items, which is a common issue in large-scale surveys. Modest reliability means comparatively high measurement noise, but this is offset by the large sample size of this study. The relatively low alpha suggests some caution regarding the interpretation of effect sizes, which may be underestimates due to the attenuating effect of measurement error.

#### 2.2.3. Early Childhood Development

##### Cognitive Development

To measure cognitive development, two subscales of the British Ability Scales Second Edition (BAS-II) were selected at 5 years of age—Pattern Construction and Picture Similarities [57]—both representing non-verbal reasoning abilities. The BAS-II is a set of standardised, age-appropriate, tests to capture cognitive ability, with suitable tests for children between 2 and 17 years of age. The Pattern Construction test assessed spatial problem-solving skills by presenting the child with a maximum of 23 items, each displaying a pattern which the child has to replicate using patterned cubes. For each item, the interviewer coded (i) whether the item was constructed correctly, and (ii) if they did so within the time limit. The patterns increased in complexity, and the assessment stopped if the child made four errors across five consecutive items. We used age- and ability-adjusted t-scores constructed using BAS-II normative data [58].

The Picture Similarities test assesses non-verbal reasoning by presenting the child with a row of four pictures or designs and asking the child to place a fifth card under the picture it best matched [57]. This task also increases in difficulty, with a maximum of 33 items. If the child made six errors across any eight consecutive items, the assessment was stopped, unless this occurred within the first eight items, in which case the child was routed back to easier items. Again, we used age- and ability-adjusted scores [58].

##### Socio-Emotional Development

Socio-emotional development at age 5 was measured using the Strengths and Difficulties Questionnaire (SDQ; [59]), completed by the primary caregiver. We used the Total Difficulties score, based on 20 items from the Emotional, Conduct, Hyperactivity, and Peer Problems subscales of the SDQ. Items are scored on a 3-point Likert scale: *Not true*, *Somewhat true*, or *Certainly true*, with *Somewhat true* always scored as 1, and the scoring of *Not true* and *Certainly true* varying per item (full scoring procedures available online: https://www.sdqscore.org, accessed on 9 April 2026). Per domain, scores ranged from 0–10, with higher scores indicating more socio-emotional problems. The maximum total score for the four domains combined was 40. Prior to hypothesis testing, the scale was recoded so that a higher score represented fewer emotional and behavioural problems and thus better socio-emotional development.

##### Language Development

Language development was measured using the Naming Vocabulary sub-test of the BAS [57]. The child was asked to name 19 items presented as pictures. If the participant made five consecutive errors, the assessment ended, unless this occurred within the first five items, in which case they were routed to easier items. Age- and ability-adjusted scores were used [58].

### 2.3. Background Variables

A set of demographic variables was included as covariates to the mediator (where applicable) and outcome variables in all analyses, covering the families’ socioeconomic status and mothers’ highest academic qualification (at age 3). Families’ socioeconomic status was measured using both OECD (Organisation for Economic Co-operation and Development) equivalised household income [60] and housing tenure (i.e., whether the main caregiver in the family was a homeowner). Educational levels were classified on a scale from *GCSE grades D-G* (1) to *Higher degree* (6).

The final set of analysis also included the parental mental health scores at age three to control for any observed effects of psychological distress during infancy that might have been driven by continuity over time.

### 2.4. Statistical Analysis

**Figure 1:**
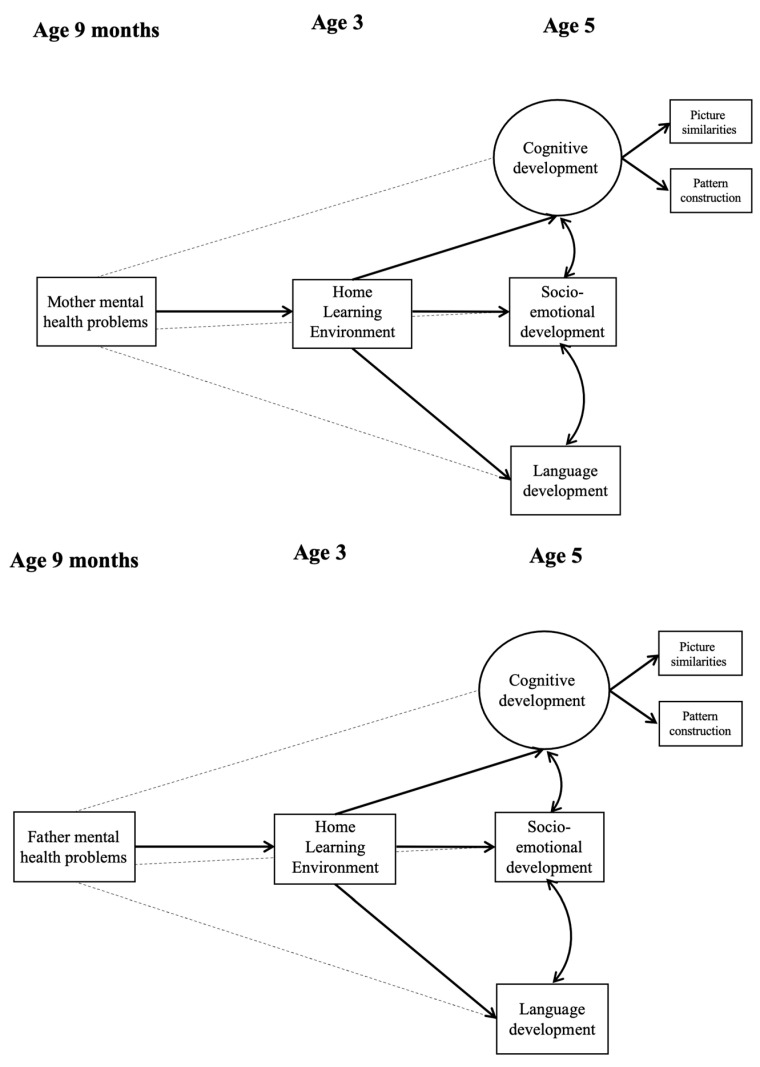
Hypothesized model with direct and indirect paths between parental mental health and child outcomes. Dashed lines represent the direct paths between parental mental health and child outcomes.

Data preparation and descriptive statistics were completed in StataNow (Version 18.5). For the descriptive analyses, the svyset command was used to account for the complex survey design by including the electoral ward, stratum ID, finite population coefficient, and the overall case weight (the product of the initial sampling weight and the attrition weight). Supplement S4 [61] provides more information on the weighting procedures that were applied in line with MCS guidelines. The main hypotheses were tested using structural equation modelling (SEM, presented in Figure 1), separately for mothers and fathers, in Mplus (version 8.11).

To test our hypotheses and assure good model fit, we built up the SEM model in three steps. In the first, we tested whether the mother’s mental health (at 9 months of age) predicted the HLE (at 3 years of age). The second model tested whether the mothers’ mental health (at 9 months of age) was associated with children’s cognitive, socio-emotional, and language development (at 5 years of age, as separate outcomes in the same model) and whether the HLE mediated these pathways. The third model was identical to the second but controlled for later (age 3) depression. Covariates representing SES indicators were added to the HLE and all child outcomes at age 5 across all models. The models described above were re-run in identical fashion for fathers’ mental health.

The indirect effects in the mediation models were calculated by multiplying the parameter estimates of the paths between parental mental health and the HLE and the HLE and each cognitive development outcome, respectively. The total effects of parental mental health on developmental outcomes were calculated by summing the direct and indirect effects. The indirect effects and their confidence intervals were estimated using bootstrapping with 1000 resamples [62].

All the models included regression paths from the observed variables to a set of covariates. Because in standard SEM models missing data in the exogenous variables leads to listwise deletion, we conducted sensitivity analyses to see whether relaxing this assumption (by treating the covariates as endogenous) affected the results. As these analyses revealed no substantive differences, the results are presented without listwise deletion.

Model fit for the structural equation models was evaluated using the following thresholds: Comparative Fit Index (CFI) > 0.90, Tucker Lewis Index (TLI) > 0.90, Root Mean Square Error of Approximation (RMSEA) < 0.08 and Standardized Root Mean Square Residual (SRMR) < 0.08 [50].

In all the SEM analyses, missing data was handled using full information maximum likelihood. The Mplus model was specified as ‘type = complex’ to incorporate information on clustering, strata, and the overall sample/attrition weights.

## 3. Results

The fit of the three models is outlined in Table 2. The standardised coefficients for direct, indirect, and total effects are included in Table 3. The full Structural Equation Model is presented in Supplementary Materials S5.

### 3.1. Maternal Mental Health, the Home Learning Environment, and Child Outcomes

As shown in Table 3, the direct association between maternal mental health at 9 months and the home learning environment at age 3 was statistically significant and in the expected negative direction (β = −0.05, *p* < 0.001). In the main model, the direct associations between maternal mental health at 9 months and children’s socio-emotional functioning (β = −0.23, 95% CI [−0.24, −0.20], *p* < 0.001) and language (β = −0.02, 95% CI [−0.05, −0.01], *p* = 0.022) development were significant and in the expected negative direction. The direct path from maternal psychological distress to children’s cognitive skills was not significant (β = −0.02, 95% CI [−0.04, −0.01], *p* = 0.22). Importantly, we found significant indirect associations between maternal mental health and child outcomes via the home learning environment. As shown in Table 3, the indirect effects of maternal mental health on children’s cognitive ability (β = −0.01, 95% CI [−0.01, −0.01], *p* < 0.001), socio-emotional functioning (β = −0.01, 95% CI [−0.01, −0.01], *p* < 0.001), and language skills (β = −0.01, 95% CI [−0.01, −0.01], *p* < 0.001) via the home learning environment were all statistically significant, though small in size. These indirect paths represented 29%, 2%, and 24% of the total effects, respectively. The proportion of the total effect represented by the indirect effect differs substantially across the developmental domains. This is largely because the beta coefficient for the direct association between the mother’s mental health and socio-emotional development was substantially larger than the other development domains.

The results of subsidiary analysis that help understanding the effects of the longevity of parental distress, controlling for maternal mental health at age 3, are shown in Table 3, Model 2. Adding maternal mental health at age three reduced the strength of the association between maternal health at 9 months and the home learning environment, although it remained significant (β = −0.03, 95% CI [−0.05, −0.01], *p* = 0.01). The direct association between maternal mental health at 9 months and children’s socio-emotional functioning (β = −0.14, 95% CI [−0.17, −0.12], *p* > 0.01) was still significant but also reduced in size. The direct associations between maternal mental health and children’s cognitive (β = −0.004, 95% CI [−0.03, 0.03], *p* = 0.81) and language abilities (β = −0.01, 95% CI [−0.03, 0.01], *p* = 0.38) were no longer significant. The indirect pathways via the home learning environment remained statistically significant for all three associations. The indirect effect of maternal mental health at 9 months on children’s cognitive ability (β = −0.004, 95% CI [−0.01, −0.01], *p* = 0.01), socio-emotional functioning (β = −0.004, 95% CI [−0.01, −0.01], *p* = 0.014), and language ability (β = −0.005, 95% CI [−0.01, −0.01], *p* = 0.01) were statistically significant but small, explaining 50%, 2%, and 33% of the total effects, respectively.

### 3.2. Paternal Mental Health, the Home Learning Environment and Child Outcomes

The SEM models were re-estimated for fathers’ mental health. Table 3 shows that fathers’ mental health at 9 months of age was significantly negatively associated with the home learning environment at age 3 (β = −0.03, 95% CI [−0.046, −0.017], *p* = 0.024). In the mediation model, paternal mental health showed significant direct associations with children’s cognitive ability (β = −0.033, 95% CI [−0.063, −0.004], *p* = 0.026) and social-emotional functioning (β = −0.081, 95% CI [−0.106, −0.054], *p* < 0.001). The direct association with children’s language ability was negative but not statistically significant (β = −0.019, 95% CI [−0.042, 0.002], *p* = 0.091). The indirect effects were small but statistically significant for cognitive development (β = −0.004, 95% CI [−0.008, −0.001], *p* = 0.025), social-emotional functioning (β = −0.004, 95% CI [−0.007, −0.001], *p* = 0.022), and language skills (β = −0.005, 95% CI [−0.010, −0.001], *p* = 0.020). These indirect pathways accounted for approximately 11%, 5%, and 21% of the total effects on cognitive development, social-emotional functioning, and language, respectively.

Finally, we also examined whether these associations remained the same when adjusting for father’s mental health at age 3. Fathers’ mental health at 9 months was no longer directly associated with the home learning environment at age 3 after contemporaneous mental health symptoms at age 3 were controlled for (β = −0.017, 95% CI [−0.046, 0.010], *p* = 0.247). The direct path to children’s social-emotional functioning remained statistically significant, although attenuated in size (β = −0.042, 95% CI [−0.077, −0.012], *p* = 0.008). In contrast, the direct associations with cognitive development (β = −0.023, 95% CI [−0.056, 0.013], *p* = 0.182) and language ability (β = −0.008, 95% CI [−0.033, 0.018], *p* = 0.518) were no longer statistically significant after adjusting for age 3 paternal mental health. The indirect pathways via the home learning environment remained consistent in direction but were no longer statistically significant. The standardized indirect effects were β = −0.003 for cognitive development (95% CI [−0.007, 0.001]), β = −0.002 for social-emotional functioning (95% CI [−0.006, 0.001]), and β = −0.003 for language ability (95% CI [−0.009, 0.001]).

## 4. Discussion

The primary aim of this study was to investigate the relationship between maternal depressive symptoms in the first year of life and the home learning environment as a potentially important and understudied mediating mechanism linking postnatal psychological distress to child developmental outcomes. We further explored the extent to which discerned associations were accounted for by later depressive symptoms, and whether similar associations are observed for fathers. To address these questions we used large-scale nationally representative data from the UK Millenium Cohort Study.

Consistent with our expectations, we found that maternal mental health problems in the first year of life were associated with the quality of the HLE at age 3. This pattern of negative associations was also replicated in the fathers’ data. Given the well documented poorer developmental outcomes of children exposed to parental depression in early life, these results are important in broadening the scope of potential parenting mechanisms that might underly these effects and the scope of potential intervention targets. The results are consistent with findings from research by Sohr-Preston and Scaramella [63], who found that parents with mental health problems tended to engage in less infant-directed speech and fewer bouts of joint attention. Contrary to Mensah & Kiernan’s [3] findings, the associations between paternal mental health and cognitive and social development remained when socioeconomic factors were controlled for.

A key hypothesis tested in these analyses was that the HLE would mediate the association between maternal (and possibly paternal) psychological distress on child development outcomes. Consistent with that, we observed significant indirect effects of maternal depression on language, cognitive, and social-emotional outcomes via the HLE. All indirect paths tended to be small in size, but statistically significant. These mediated pathways were replicated for fathers. These findings support the interpretation that parental mental health symptoms are associated with fewer enriching learning activities, which in turn negatively affect child outcomes. This suggests that Kiernan and Huerta’s [39] finding that children of mothers with higher depression scores were read to less often is not restricted to reading and includes a wider range of activities that stimulate child development. Although these findings are consistent with the interpretation that parental distress causes reductions in home learning interactions, it is important to recognize that our analyses are correlational and cannot directly establish this. It is possible, for example, that parental distress and caregiving interactions such as the home learning environment influence each other, as negative parent–child interactions can impact parental mood.

Notably, we found that the mediating pathways from parental distress to early childhood outcomes were more statistically robust for mothers than fathers. This difference could be due to a number of factors, including lower power (there were fewer fathers in this study than mothers) or the fact that mothers were most often the reporters of the home learning environment. While the use of a single reporter for maternal distress and the HLE raises the possibility of common method variance, prior research shows that independently reported maternal and paternal engagement in cognitively stimulating activities predicts directly assessed child cognitive outcomes [63], supporting the validity of parent-reported home learning measures. Furthermore, different parental responsibilities may explain why the magnitude of the effect for fathers was smaller than that for mothers. Mothers provide almost double the amount of childcare compared to fathers [64], which may contribute to why associations between fathers’ mental health and the HLE were lower in magnitude. Another important possibility, consistent with previous research, is that paternal distress impacts child development more indirectly, for example, through its impact on the couple relationship [65].

In follow-up analyses, we explored whether the indirect pathways between psychological distress at 9 months, the HLE at age 3, and outcomes at age 5 remained after controlling for depression at 3 years. Maternal mental health at age 3 was moderately correlated with earlier symptoms, and its inclusion substantially reduced the effects of mental health at 9 months on both the HLE and child outcomes. This pattern was replicated with fathers: mediation was observed when not controlling for age 3 depression but was substantially reduced when it was (indeed, mediation became non-significant for others across all outcomes). This pattern suggests that later parental mental health exerts a substantial influence on children’s developmental outcomes, and that continuity in depressive symptoms may substantially or even fully (in the case of fathers) account for the association between postnatal depressive symptoms, the home learning and environment and child outcomes at age 5. This is consistent with wider evidence that the timing and chronicity of parental depression play an important role in shaping its effects on child development [66]. However, it is important to note that even after controlling for age 3 depression, the indirect pathways via the HLE remained significant for mothers, though not for fathers. This suggests that for mothers at least, there may be some unique contribution of psychological distress in infancy to later childhood outcomes.

These findings suggest a number of potentially important avenues for future research. In particular, they highlight the importance of going beyond broad household-level measures of the HLE to examine more precisely which positive parenting behaviours might be affected by parental mental health difficulties, whether these processes differ for mothers and fathers, and how those behaviours could be appropriately targeted in interventions. This may be especially relevant for fathers, as paternal associations observed in this study were smaller and less consistent. The HLE Index, measuring the frequency of engaging in certain activities with the child on a household level, might not be sensitive to the different ways in which fathers contribute to parenting, which can qualitatively differ from mothers’ behaviour [67].

### Limitations and Future Directions

Although the current analyses have notable strengths, particularly the large-scale nationally representative data and the objective assessments of development conducted at age 5, this study has several limitations that are important to consider when interpreting our findings. Some of these limitations concern measurement. The HLE Index is a parent self-report measure, which raises important questions about the possibility of shared method bias. The reliance on parent report for both depressive symptoms and the HLE means that the findings reported herein should be treated with caution. This is a particular concern for the maternal data where the same reporter rated psychological distress symptoms and the HLE, risking common method variance. We cannot rule out potential measurement confounds here. It is notable though that for mothers, the mediating pathways we estimated between psychological distress in infancy and child outcomes via the HLE remained when parent-reported depression symptoms at age 3 were controlled for. If common method variance was solely responsible for the associations, this would not be expected. An additional limitation of relying on a single rating of the home learning environment frequency is that it does not capture the parenting behaviours of specific adults. Thus, the observed associations between individual parents’ reports of depressive symptoms and the home learning environment should be thought of as reflecting the contributions of each parent’s depressive symptoms to the child’s overall home learning environment, rather than the parent’s specific behaviours. In addition, the HLE is unlikely to reflect a purely environmental construct, as genetic research suggests that characteristics of both parents and children are associated with the quality of the HLE [68]. Therefore, some observed associations may partly reflect genetic confounding of the parents’ and child’s genes or a gene–environment interaction. Moreover, parents’ responses may have been influenced by social desirability, leading to potential over-reporting for the HLE or under-reporting for both the Rutter Malaise Inventory and the SDQ. Finally, the modest internal consistency of the HLE Index suggests that the index contains considerable measurement error. Furthermore, studies have shown that observed interactions between parent and child were better predictors of child outcomes than parent reports [69]. Therefore, capturing the HLE through multi-informant reports or observational methods in future studies would facilitate a better understanding of home learning environment provision and its role in child outcomes.

The limitations described above are particularly difficult to address in large-scale studies, but recent methodological and technological advances make them increasingly surmountable. While parent-reported data remain valuable, they are, ideally, complemented by measures that provide a deeper and more objective insight into the quality of parent–child interactions. Emerging technologies offer promising avenues for this. For example, systems such as OpenPose can rapidly detect key points of the human body, face, hands, and feet in real time, allowing researchers to infer facial expressions—for instance, raised eyebrows for yes/no questions or surprise, and lowered brows for anger [70,71]. This type of automated analysis could support scalable assessments of interaction quality. Similarly, language environment analysis tools (e.g., LENA) use wearable recording devices to capture and algorithmically analyse the speech environment around children over extended periods of time, providing another scalable method for assessing parent–child interactions [72].

## 5. Conclusions

This study highlights the potential role that maternal and paternal mental health play in young children’s developmental outcomes, both directly and indirectly through the home learning environment. These findings emphasise the importance of early parental mental health support, while also indicating a need for further research into which specific parenting behaviours are most affected by parental distress and how such pathways might best inform intervention.

## Figures and Tables

**Table 1:** Ethnicity of Cohort members, mothers and fathers.

	Cohort Member	Mother	Father
White	86.55%	88.87%	90.66%
Pakistani	3.23%	3.13%	2.44%
Mixed	3.21%	0.88%	0.68%
Indian	1.87%	0.19%	2.24%
Black African	1.59%	1.54%	0.86%
Black Caribbean	1.05%	1.14%	0.89%
Bangladeshi	1.03%	0.96%	0.66%
Other Ethnic group	1.49%	1.57%	1.57%
Unweighted base	15,607	14,722	8767
Weighted base	16,114	15,096	9220

**Table 2:** Model fit statistics for Models 1 and 2.

Model	*X* ^2^	*df*	*p*	CFI	TLI	RMSEA (90% CI)
*Mother’s mental health*						
Model 1: MMH → HLE → Outcomes	8291.65	33	<0.001	0.99	0.99	0.01 (0.00–0.02)
Model 2: control for MMH age 3	10,921.25	42	<0.001	0.99	0.99	0.01 (0.00–0.02)
*Father’s mental health*			<0.001	1.00	1.00	0.00 (0.00–0.00)
Model 1: FMH → HLE → Outcomes	7633.795	30	<0.001	0.99	0.99	0.01 (0.00–0.02)
Model 2: control for FMH age 3	7723.929	35	<0.001	0.99	0.99	0.01 (0.00–0.02)

**Table 3:** Standardised coefficients and standard error for Models 1and 2.

	*Mothers*		*Fathers*	
	Model 1	Model 2 ^+^	Model 1	Model 2 ^+^
	Std. Est. (SE)	Std. Est. (SE)	Std. Est. (SE)	Std. Est. (SE)
*Mental health* → *Home learning* *environment*	−0.05 (0.01) ***	−0.03 (0.01) *	−0.03 (0.02) *	−0.02 (0.2)
*Mental health* → *Cognitive development*				
Total	**−0.02 (0.01)**	−0.008 (0.02)	−0.04 (0.02) *	−0.03 (0.02)
Direct	**−0.02 (0.01)**	−0.004 (0.002)	−0.03 (0.02) *	−0.02 (0.02)
Via HLE	−0.007 (0.002) ***	−0.004 (0.001) *	−0.004 (0.002) *	−0.001 (0.002)
*Mental health* → *Socio-emotional development*				
Total	−0.23 (0.01) ***	−0.15 (0.01) ***	−0.08 (0.01) ***	−0.045 (0.02) **
Direct	−0.23 (0.01) ***	−0.14 (0.01) ***	−0.08 (0.002) ***	−0.04 (0.002) **
Via HLE	−0.006 (0.001) ***	−0.004 (0.002) *	−0.004 (0.002) *	−0.002 (0.002)
*Mental health* → *Language development*				
Total	−0.03 (0.01) **	−0.02 (0.01)	−0.02 (0.01) *	−0.01 (0.01)
Direct	−0.02 (0.01) *	−0.01 (0.01)	−0.02 (0.01) *	−0.008 (0.01)
Via HLE	−0.008 (0.002) ***	−0.005 (0.002) **	−0.005 (0.002) *	−0.003 (0.003)

*Note.* Statistical significance * *p* < 0.05, ** *p* < 0.01, *** *p* < 0.001. The models include 1000 bootstraps. ^+^ Model controls for mental health at age 3.

## Data Availability

The data for the Millennium Cohort Study are deposited in the UK Data Archive, University of Essex. The code that supports the findings of this study is available from the corresponding author, upon reasonable request.

## Supplementary Materials

The following supporting information can be downloaded at: https://www.mdpi.com/article/10.3390/children13050582/s1, Supplement S1: Sampling strategy and dataset; Supplement S2: Confirmatory Factor Analysis for Mothers’ and Fathers’ Rutter Malaise Inventory; Table S1: Confirmatory Factor Analysis of Parental Mental Health; Supplement S3: Home learning environment index; Supplement S4: Weighting; Supplement S5: Direct and indirect effects of parental mental health on child outcomes. Figure S1: Direct and indirect effects of parental mental health on child outcomes.

## Abbreviations

The following abbreviations are used in this manuscript:

CFA	Confirmatory Factor Analysis
HLE	Home learning environment
HMRC	His Majesty Revenues and Customs
MCS	Millennium Cohort Study
OECD	Organisation for Economic Co-operation and Development
SES	Socioeconomic status
