# Supplementary material for: The Home Learning Environment as a Mediator of the Impact of Parental Psychological Distress on Child Development
Source: Children (Basel). 2026 Apr 22;13(5):582. doi: 10.3390/children13050582 (PMC13204149; doi:10.3390/children13050582)
Supplement: Supplementary file 1 [file children-13-00582-s001.zip › children-4223504-supplementary.pdf]

children

MDPI

Supplement

The Home Learning Environment as a Mediator of the Impact of Parental Psychological Distress on Child Development

Marie-Louise (Jessica) A. J. van de Grint-Stoop [1,*], Laurel A. Fish [2], Chloe Austerberry [1], Marialivia Bernardi [2] and R. M. Pasco Fearon [1,2,*]

[1] Centre for Child, Adolescent and Family Research, Department of Psychology, University of Cambridge, Rayleigh Wing, Free School Lane, Cambridge CB2 3RF, UK
[2] Department of Clinical, Educational and Health Psychology, University College London, Gower Street, London WC1E 6BT, UK
* Correspondence: mlajv2@cam.ac.uk (M.-L.A.J.v.d.G.-S.); pf380@cam.ac.uk (R.M.P.F.)

Supplementary Materials

S1 Sampling Strategy and Dataset

In the initial sample design, families from areas with high poverty rates and from ethnic minority backgrounds were intentionally over-represented to account for expected non-response and attrition. More detailed information about the sampling strategies can be found in the MCS data guide [45]. Full documentation on the survey is available within the data deposited in the UK Data Archive, University of Essex [46].

S2 Confirmatory Factor Analysis Parental Mental Health

Prior to the main analyses, we performed two confirmatory factor analyses of the items measuring parental mental health problems. Model fit was evaluated against the following thresholds for good model fit: Comparative Fit Index (CFI) > 0.90, Tucker Lewis Index (TLI) > 0.90, Root Mean Square Error of Approximation (RMSEA) < 0.08, and Standardized Root Mean Square Residual (SRMR) < 0.08 [50]. Supplementary Table 1 shows the standardised factor loadings and standard errors for both CFAs.

A confirmatory factor analysis (CFA) was first conducted on the individual Rutter Malaise Inventory items to confirm their measurement structure for maternal and paternal symptom factors, with the results shown in Supplementary Table 1. The model demonstrated good fit, $\chi^2(210) = 18{,}520.93$, $p < .001$; CFI = .96; TLI = .95; RMSEA = .04, 90% CI [.03, .04]; SRMR = 0.03. Standardized loadings ranged from .28 to .66 (all $p < .001$). Although some of the loadings were moderate in magnitude, this might be due to the binary nature of the inventory, in which case the lower loadings more likely reflect heterogeneous content than measurement weakness [51]. To preserve conceptual breadth and comparability with prior applications of the Rutter Malaise Inventory, we retained all items for the second CFA.

S3 Home Learning Environment Index

The HLE Index was developed and validated using the EPPSE and Growing Up in Scotland studies [27, 55]. For all the items in the scale, parents report whether and how often anyone in the household undertakes the specific activities with their child. At the time of data collection for the Millennium Cohort Study (MCS) age 3, the HLE Index had not been validated as such, but all questions were included in the survey [56]. As a consequence, the HLE has not been widely studied using data from the MCS.

Academic Editor: Cristina Nunes

Received: 11 March 2026
Revised: 12 April 2026
Accepted: 17 April 2026
Published: 22 April 2026

Copyright: © 2026 by the authors. Submitted for possible open access publication under the terms and conditions of the Creative Commons Attribution (CC BY) license.

Table S1. Confirmatory Factor Analysis for Mothers' and Fathers' Rutter Malaise Inventory

		Std. Est.	*SE*
**CFA 1**	**Mothers' Rutter Malaise Inventory**		
	Do you feel tired most of the time?	0.392	0.010
	Do you often feel miserable or depressed?	0.622	0.010
	Do you often get worried about things?	0.514	0.010
	Do you often get into a violent rage?	0.296	0.018
	Do you often suddenly become scared for no good reason	0.474	0.015
	Are you easily upset or irritated?	0.621	0.011
	Are you constantly keyed up and jittery?	0.475	0.017
	Does every little thing get on your nerves and wear you out?	0.527	0.015
	Does your heart often race like mad?	0.452	0.017
	**Fathers' Rutter Malaise Inventory**		
	Do you feel tired most of the time?	0.343	0.013
	Do you often feel miserable or depressed?	0.556	0.014
	Do you often get worried about things?	0.471	0.014
	Do you often get into a violent rage?	0.389	0.025
	Do you often suddenly become scared for no good reason	0.420	0.020
	Are you easily upset or irritated?	0.541	0.011
	Are you constantly keyed up and jittery?	0.484	0.019
	Does every little thing get on your nerves and wear you out?	0.533	0.019
	Does your heart often race like mad?	0.477	0.019

Note. Model Fit Statistics: CFA1: $\chi^2(210) = 18{,}520.93$, CFI = 0.96, TLI = 0.95, RMSEA = 0.04 (95% CI = 0.03, 0.04), SRMR = 0.03.

S4 Weighting

All analyses in this paper used weights to adjust for sampling probabilities and the likelihood of attrition or non-response. Therefore, the frequencies and analyses presented here represent population estimates. Sampling weights were combined with attrition/non-response weights to create an overall weight. Weighted and unweighted bases are reported in the tables for descriptive purposes. The weight applied was the overall weight (including both the sampling and attrition weights) based on the most recent sweep in which the family participated, as specified by MCS guidance. Further details on sampling and non-response weights can be found in the MCS 'Study User Guide to Analysing MCS Data Using STATA' [61].

S5 Direct and Indirect Effects of Parental Mental Health on Child Outcomes

Figure S1. Direct and indirect effects of parental mental health on child outcomes.

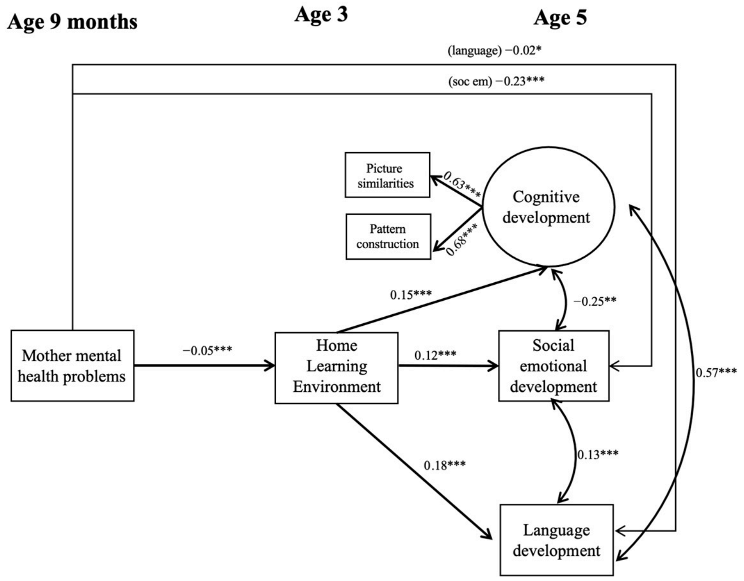

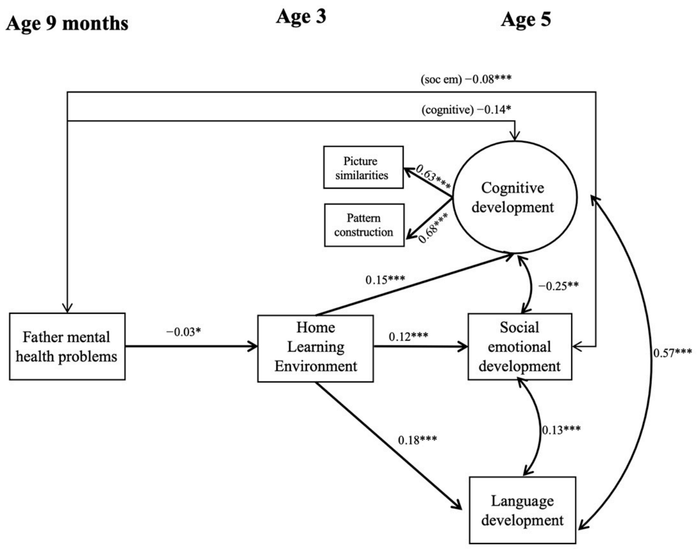

Note. Statistical significance: **p* < 0.05, **p < 0.01, ***p < 0.001.
Only significant associations are included.
This model includes control variables (housing tenure, OECD equivalised income, and mother's academic qualification) for all dependent variables.

Abbreviations

The following abbreviations are used in this manuscript:

HLE	Home learning environment
CFA	Confirmatory Factor Analysis

Disclaimer/Publisher's Note: The statements, opinions and data contained in all publications are solely those of the individual author(s) and contributor(s) and not of MDPI and/or the editor(s). MDPI and/or the editor(s) disclaim responsibility for any injury to people or property resulting from any ideas, methods, instructions or products referred to in the content.
